# Development and Validation of a Plasma FAM19A5 and MRI-Based Radiomics Model for Prediction of Parkinson’s Disease and Parkinson’s Disease With Depression

**DOI:** 10.3389/fnins.2021.795539

**Published:** 2021-12-17

**Authors:** Xue-ning Li, Da-peng Hao, Mei-jie Qu, Meng Zhang, An-bang Ma, Xu-dong Pan, Ai-jun Ma

**Affiliations:** ^1^Department of Neurology, The Affiliated Hospital of Qingdao University, Qingdao, China; ^2^Department of Radiology, The Affiliated Hospital of Qingdao University, Qingdao, China; ^3^Shanghai Xunshi Technology Co., Ltd., Shanghai, China; ^4^Institute of Cerebrovascular, The Affiliated Hospital of Qingdao University, Qingdao, China

**Keywords:** Parkinson’s disease, Parkinson’s disease with depression, plasma FAM19A5, radiomics, machine learning

## Abstract

**Background:** Prediction and early diagnosis of Parkinson’s disease (PD) and Parkinson’s disease with depression (PDD) are essential for the clinical management of PD.

**Objectives:** The present study aimed to develop a plasma Family with sequence similarity 19, member A5 (FAM19A5) and MRI-based radiomics nomogram to predict PD and PDD.

**Methods:** The study involved 176 PD patients and 181 healthy controls (HC). Sandwich enzyme-linked immunosorbent assay (ELISA) was used to measure FAM19A5 concentration in the plasma samples collected from all participants. For enrolled subjects, MRI data were collected from 164 individuals (82 in the PD group and 82 in the HC group). The bilateral amygdala, head of the caudate nucleus, putamen, and substantia nigra, and red nucleus were manually labeled on the MR images. Radiomics features of the labeled regions were extracted. Further, machine learning methods were applied to shrink the feature size and build a predictive radiomics signature. The resulting radiomics signature was combined with plasma FAM19A5 concentration and other risk factors to establish logistic regression models for the prediction of PD and PDD.

**Results:** The plasma FAM19A5 levels (2.456 ± 0.517) were recorded to be significantly higher in the PD group as compared to the HC group (2.23 ± 0.457) (*P* < 0.001). Importantly, the plasma FAM19A5 levels were also significantly higher in the PDD subgroup (2.577 ± 0.408) as compared to the non-depressive subgroup (2.406 ± 0.549) (*P* = 0.045 < 0.05). The model based on the combination of plasma FAM19A5 and radiomics signature showed excellent predictive validity for PD and PDD, with AUCs of 0.913 (95% CI: 0.861–0.955) and 0.937 (95% CI: 0.845–0.970), respectively.

**Conclusion:** Altogether, the present study reported the development of nomograms incorporating radiomics signature, plasma FAM19A5, and clinical risk factors, which might serve as potential tools for early prediction of PD and PDD in clinical settings.

## Introduction

Parkinson’s disease (PD) is the second most common neurodegenerative disease in the world. The main pathological changes reported in PD include degeneration of dopaminergic neurons in the substantia nigra pars compacta, decreased striatal dopamine, and neuronal formation of Lewy bodies (Jankovic, 2008). Clinical diagnosis of PD is often based on the emergence of motor symptoms. However, certain studies have shown that in many cases non-motor symptoms are often present before the onset of motor symptoms (Schapira et al., 2017). Therefore, non-motor symptoms are potentially more valuable for the early PD diagnosis. The onset of depression seems to have no clear correlation with the course of PD. It might appear during the early or late stages of the disease. Previous studies have shown that atrophy occurs in the putamen, globus pallidus, dorsal thalamus, midbrain, and other regions of the brain in PD patients with depression (PDD) (Shiba et al., 2000). It is expected that treatment during the early stages of the disease would improve depressive symptoms. In fact, some of the studies considered depression to be the strongest predictor of impaired quality of life in PD patients (Benoit and Robert, 2011). Therefore, it is important to recognize the risk of depression during the early stages of PD.

Neuroinflammation occupies a pivotal position in the pathogenesis of PD (Cardoso et al., 2009; Yu et al., 2020). In fact, both the release of numerous pro-inflammatory mediators and activation of immune cells are known to be involved in the regulation of neurogenesis, reduction of synaptic plasticity, and neuronal survival. These in turn reduce the binding of receptors, present on the surface of neurons, to excitatory neurotransmitters, which affect emotions negatively (Hu et al., 2015). Immune cells, such as astrocytes, not only exist in all the areas of the brain but these cells are positioned in close proximity to neuronal structures (Prange et al., 2019). This ensures direct communication between the cells. Under pathological conditions, astrocytes release pro-inflammatory factors, such as IL-1, IL-6, and TNF-α, and chemokines such as CCL2, CCL3, CCL5, and CCL8. All these molecules can damage dopaminergic neurons, when present in excessively elevated levels. In fact, these factors can also interact with the receptors present on the surface of microglia and glutamatergic neurons, inducing a cascade of neuroinflammatory reactions (van Mierlo et al., 2015). In the Central Nervous System (CNS), microglial cells secrete glutamate and are metabolized to quinolinic acid by kynurenine. In this case, astrocytes can enhance neuronal survival *via* glutamate uptake, consequently, released glutamate once exceed the amount that could be cleared by astrocytes *via* reuptake (Choudary et al., 2005; Pierozan et al., 2016). Together, glutamate and quinolinic acid will further enhance neurotoxicity, leading to the development of depressive symptoms (Ali et al., 2011). In recent times, FAM19A5 was defined as a new type of chemokine. This is mainly attributed to its similarity to the CC chemokine family, in terms of nature, and induction of reactive astrocytosis during immune activation, following CNS injury. Theoretically, it could be utilized as an immunoreactive brain-specific chemokine. In fact, it has been previously reported to control axonal sprouting and functional recovery after brain injury (Dias et al., 2013). Recent studies showed that increased FAM19A5 expression promoted major depression (Burda and Sofroniew, 2014). The occurrence and development of vascular dementia in patients with severe depression linked increased plasma FAM19A5 levels to cortical atrophy (Pekny and Nilsson, 2005). However, associations between FAM19A5 and depression have not been previously studied in PD.

Radiomics analysis is a process that is used to extract quantitative features from medical images *via* advanced feature extraction procedures (Arnone, 2020). Subsequently, machine learning methods, such as the least absolute shrinkage and selection operator LASSO) logistic regression method was used to shrink the dimension of radiomics features, and build models for disease detection and classification, prognosis prediction, and therapeutic response evaluation (Rappold and Tieu, 2010). Radiometric is widely used to predict the recurrence of many cancer diseases, such as optic neuroblastoma, colorectal cancer, and liver cancer (Ammari et al., 2020; Antunes et al., 2020; Li Z. et al., 2020; Zhao et al., 2021), which inspire us. During post-mortem analyses of the brain of PD patients, astrocyte density was found to be reduced in the substantia nigra, and the severity of dopamine neuronal loss was positively correlated with astrocytic α-synuclein (Carta et al., 2017). Neuroimaging studies also confirmed these findings. Recent imaging omics studies showed that certain inflammatory factors were associated with reduced gray matter volume (Harms et al., 2018). However, no previous studies reported the utilization of radiomics and machine learning studies, combined with plasma FAM19A5 for the prediction of PD and PDD.

The present retrospective study was based on the hypothesis that increased blood FAM19A5 would reflect neuroinflammation and neurodegeneration in PD patients, and thus plasma FAM19A5 levels in PD patients are related to depressive symptoms. The present study aimed to create prognostic logistic regression models based on plasma FAM19A5 levels, radiomics signature, and other clinical risk factors, to predict the occurrence of PD and PDD, which would further provide a valuable tool for early diagnosis of PD and PDD in clinical settings.

## Materials and Methods

### Participants

The present study protocol received ethical approval from the Ethics Committee of the Affiliated Hospital of Qingdao University. All participants agreed to provide their written informed consent to participate in the study. Participants with idiopathic PD were diagnosed by neurologists with reference to the clinical criteria formulated by the Movement Disorders Society (Hirsch et al., 2012; Postuma et al., 2015). A total of 176 individuals with PD and 181 healthy controls were enrolled in this study from May 2018 to September 2020. The patient exclusion criteria were as follows: (1) patients with Parkinson’s plus syndrome (such as multiple system atrophy, progressive supranuclear palsy, or corticobasal degeneration) and any type of secondary Parkinson’s syndrome; (2) severe complications or insufficiency of the heart, brain, liver, and kidney. For comparison, 181 age- and sex-matched individuals were included in the HC group. The inclusion criteria for the HC group were as follows: no obvious abnormalities detected during routine physical examination, no history of CNS disease, no history of long-term use of drugs affecting neurological diseases, and no family genetic history of PD.

### Clinical Data Evaluation and Grouping

Retrospective analyses were conducted using the clinical data of all patients, including the age of onset, gender, non-motor symptoms, duration of disease, age, levodopa daily equivalent dose (LEDD) (Tomlinson et al., 2010; Köhler et al., 2017), Patient Health Questionnaire-9 (PHQ-9) (Spitzer et al., 1999; Williams et al., 2012; Chagas et al., 2013; Hurley and Tizabi, 2013), the modified Hoehn and Yahr (H&Y) scale (Tom Tang et al., 2004), and UPDRS Part III (motor symptoms) score. The H&Y scale established the stage of PD: early stage 1–2 or moderately advanced stage 2.5–5 (Goetz et al., 2004; Kang et al., 2005; Kahn et al., 2012; Brys et al., 2016). According to the standard of the UPDRS Part III (motor symptoms), the tremor score was calculated as the sum of UPDRS III items 20 and 21 divided by 7, while the non-tremor score was calculated by summing the UPDRS III items 18, 19, 22, and 27–31 divided by 12 (Han et al., 2020). If the patient’s average tremor score was more than 2 times the average non-tremor score, the condition was defined as tremor-dominant (TD). If the patient’s average non-tremor score was more than 2 times the average tremor score, it was defined as akinetic-rigid (AR). The remaining patients with the difference between tremor and non-tremor scores < 2x were categorized to have a mixed subtype (MT) condition (Rajput et al., 2017; Li J. et al., 2020). Statistics of 3 non-motor symptoms of PD patients were compared in a subgroup analysis: (1) Constipation—according to the presence of gastrointestinal symptoms as measured by ROME III functional constipation criteria (Kumar et al., 2012; Knudsen et al., 2017), subjective constipation was defined by > 25% of bowel movements being characterized by two or more symptoms: (i) straining during defecation, (ii) lumpy/hard stools, (iii) the sensation of incomplete evacuation, (iv) the sensation of anorectal obstruction, (v) manual maneuvers to assist defecation, or (vi) < 3 bowel movements in a week. According to the ROME III criteria, patients were divided into constipation and a non-constipation group; (2) Rapid eye movement (REM) sleep behavior disorder (RBD)—patients with a score of ≥ 6 and < 6 on the Rapid Eye Movement Sleep Behavior Disorder Screening Scale (RBDSQ) (Nomura et al., 2011; Gillies et al., 2016) were categorized into a PD with RBD group and a PD without RBD group, respectively; (3) Depression–patients with a score of ≥ 10 and < 10 on the PHQ-9 depression scale were categorized into a PDD group and a PD without depression group, respectively (Chaddad et al., 2019).

### Measurement of the Plasma FAM19A5 Levels

All subjects fasted for > 8 h before an early morning blood collection *via* the cubital veins. The venous blood samples were collected in ethylene diamine tetraacetic acid (EDTA) tubes and dispatched to the Inspection Department at the Institute of Cerebrovascular, Qingdao University Affiliated Hospital for further analyses. Briefly, the blood sample was centrifuged at 1,000 × *g* for 15 min to obtain two layers (Thermo Fisher Scientific, Am Kalkberg, Germany). The upper layer of transparent light-yellow liquid, which represented the plasma, was collected and frozen at –80°C. According to the manufacturer’s instructions. the plasma FAM19A5 levels were determined using 50 μL of each sample by the FAM19A5 ELISA kit (Jiang Lai, Shanghai, China). The absorbance was measured at 450 nm by using the SpectraMax M4 Multiplate Reader (Molecular Devices, Wokingham, United Kingdom).

### MRI Acquisition

MRI examination was performed within 24 h of venous blood collection. The subjects who met this criterion (82 PD patients and 82 healthy controls) were selected for routine MRI scans using the GE Signa 1.5 T and 3.0 T MRI scanners (General Electric Healthcare, Milwaukee, WI, United States) with an 8-channel phased-array head coil. The number of patients with 1.5-T scanners in the HC group was 40, while that of patients with 3.0-T scanners in the HC group was 42. In the PD group, there were 54 MRI images with a 1.5-T field strength and 28 with a 3.0-T field strength. The parameters of a three-dimensional T1-weighted brain scan were as follows: TR-550–2,200 ms, TE-2.5–33 ms, FA-69∼111°, FOV-22—24 cm, and 5-mm slice thickness. The parameters of a three-dimensional T2-weighted brain scan included: TR-3,400—6,554 ms, TE-90–150 ms, FA-90—150°, FOV- 22—24 cm, and 5-mm slice thickness. The parameters of the T2 liquid attenuation inversion recovery sequence scan were as follows: TR-6,004–9,000 ms, TE-85—154 ms, FA-90—160°, FOV-22—24 cm, and 5-mm slice thickness.

### Neuroimage Processing

#### Manually Segmentation of the Region of Interest

**FIGURE 1:**
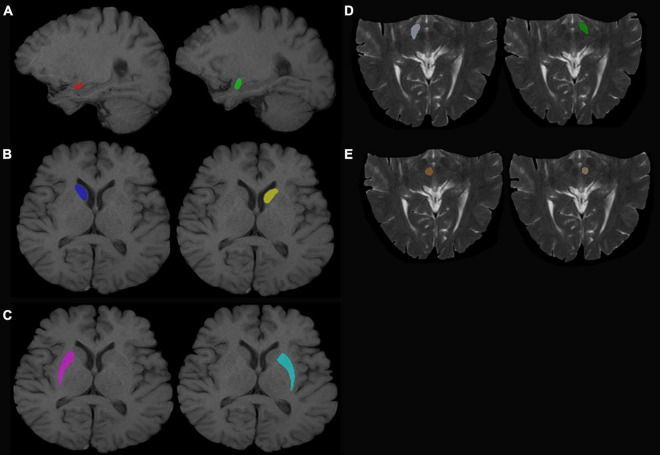
Regions of interest. **(A)** Left (green) and right (red) amygdala. **(B)** Left (yellow) and right (dark blue) caudate nucleus head. **(C)** Left (sky blue) and right (purple) putamen. **(D)** Left (dark green) and right (white) SN. **(E)** Left (beige) and right (red) red nucleus.

T1-weighted (T1W) images and T2-weighted (T2W) images were manually labeled using ITK-SNAP (Huang et al., 2016; Wilson et al., 2019)^1^ by three experienced radiologists. The labeled ROI in the T1W image included the bilateral amygdala, bilateral caudate nucleus head, and bilateral putamen. The labeled ROI in T2W images included the bilateral substantia nigra and red nucleus (Figure 1, for example, images).

#### Radiomics Feature Extraction and Analysis

**FIGURE 2:**
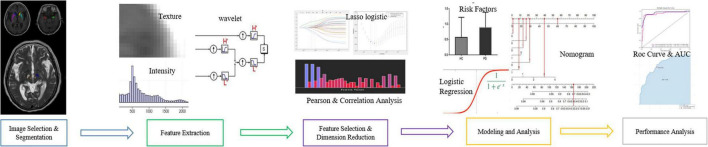
Workflow chart for radiomics feature extraction and analysis.

The workflow of radiomics feature extraction and prediction modeling is shown in Figure 2. In the feature extraction procedure, all Digital Imaging and Communications in Medicine (DICOM) formatted images were resampled with the same pixel spacing (0.5 × 0.5 mm). The Pyradiomics toolkit^2^ was utilized to extract 322 radiomics features for each ROI, including 16 shape and size features related to the three-dimensional size and shape of the ROI; 18 first-order features based on the distribution of voxel intensities; 104 texture-based features, which were calculated from the gray-level co-occurrence matrix (GLCM), gray-level run-length matrix (GLRLM), gray-level size-zone matrix (GLSZM), and gray-level dependence matrix (GLDM)—the above features have been proven to showcase the characteristics of cancer heterogeneity and potentially reflect changes in the image structure (Mayerhoefer et al., 2020); and 184 wavelet features, which were decompositions of first-order statistics and texture features. Finally, there were a total of 1,610 radiomics features extracted from 10 ROIs defined on T1W and T2W extracted for each subject. In the feature selection and feature dimension reduction procedure, Pearson correlation analyses and multivariate analyses were employed to eliminate poorly correlated and repeated radiomics features. LASSO logistical regression was performed to further shrink the effective radiomic feature dimension and normalize the radiomics features to 0–1. The normalized radiomics feature is the radiomics signature.

### Parkinson’s Disease Prediction Model

**FIGURE 3:**
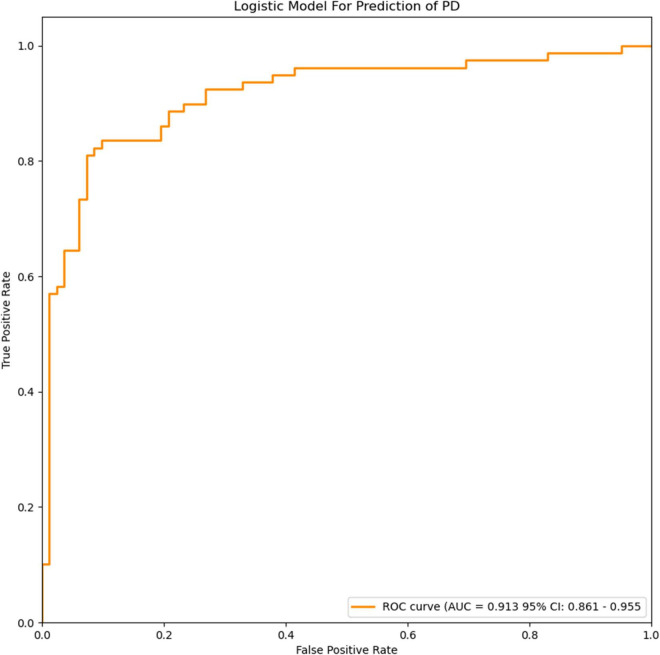
ROC curves of the logistic model for prediction of PD. The accuracy of the logistic model in predicting PD was 85.6% (AUC = 0.913, 95% CI = 0.861–0.955).

The radiomics signature extracted from bilateral substantia nigra and red nucleus regions, the plasma FAM19A5 level, gender, and age were included as risk factors to predict the PD diagnosis with logistic regression. Then, fivefold cross-validation was conducted to test the performance of the logistic regression model after calculating the ROC curve and AUC value. The prediction model was visualized using a nomogram (Figure 3).

### Parkinson’s Disease With Depression Prediction Model

**FIGURE 4:**
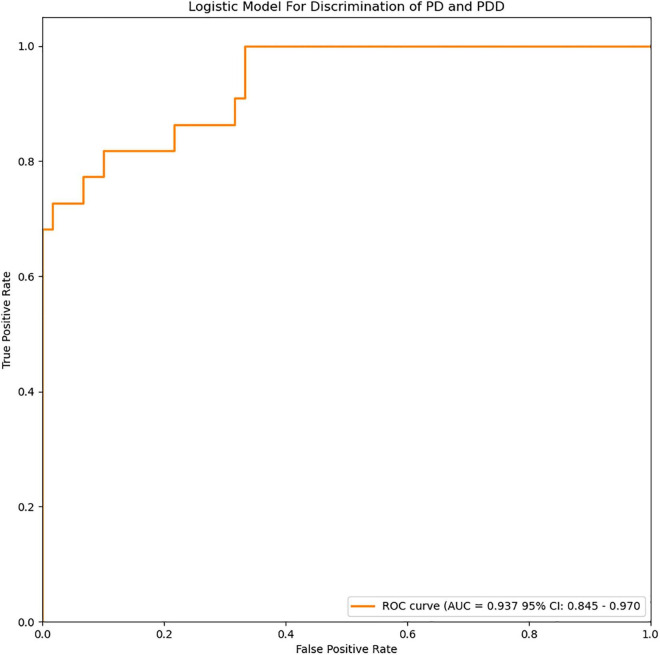
ROC curves of the logistic model for discrimination of PD and PDD. The accuracy of the logistic model in predicting PDD was 87.8% (AUC = 0.937, 95% CI = 0.845–0.970).

The radiomics signature extracted from the bilateral amygdala, head of the caudate nucleus, putamen, the plasma FAM19A5 level, gender, and age were included as risk factors to predict PDD diagnosis with logistic regression. Then, fivefold cross-validation was performed to test the performance of the logistic regression. The ROC curves were drawn to visualize the ability of the logistic model to identify the PD depressive and PD without depressive subgroups. The logistic model was visualized using a nomogram (Figure 4).

### Statistical Analysis

The experimental data were statistically analyzed using the SPSS 26 software; the plasma FAM19A5 levels were converted to a normal distribution by natural logarithm (log-transformed plasma FAM19A5 levels) and then analyzed. The gender distribution of the two groups was compared by the Chi-squared test. Student’s *t*-tests or Mann-Whitney U-tests were performed for between-group comparisons. Measurement data were expressed as mean ± standard deviation (SD). Kruskal—Wallis test and Chi-squared test were performed to compare the AR, TD, and MT groups. ANCOVA was performed to assess group differences in the plasma FAM19A5 levels, with grouping as a fixed factor and age and gender as covariates. The threshold of statistical significance was set to *P* < 0.05.

## Results

### Comparison of Baseline Data and Plasma FAM19A5 Levels Between Parkinson’s Disease Group and Healthy Control Group

No significant differences were recorded between PD and HC groups in terms of age or gender (Table 1). Interestingly, log-transformed plasma FAM19A5 levels were found to be significantly higher in the PD group as compared to the HC group (*P* < 0.001, *t* = –4.370). There were no significant correlations between plasma FAM19A5 and gender or age.

**TABLE 1:** Demographic and clinical characteristics of patients with Parkinson’s disease and healthy controls.

	PD	HC	Test value	*P*-value
Number	176	181		
Age (years)	67.35 ± 9.50	65.57 ± 10.25	*t* = 1.698	0.090
Gender (female/male)	102/79	104/72	χ^2^ = 0.274	0.601
FAM19A5(log)	2.46 ± 0.51	2.23 ± 0.46	*t* = 4.433	<0.001*
Modified HY-stage	2 (1.4)	/	/	/
Duration of illness (months)	24 (12.60)	/	/	/
LEDD	337 (237.375)	/	/	/

*Normally distributed data are presented as the means ± standard deviation (SD), skewed data are presented as the median (interquartile range), and categorical data are presented as the count (percentage). FAM19A5(log), log-transformed plasma levels of FAM19A5; LEDD, levodopa daily equivalent dose.*

* indicates statistically significant values with P < 0.05.

### Expression of Plasma FAM19A5 in Motor Subtypes

According to UPDRS Part-score, 47 patients were included in the akinetic-rigid group (AR), 71 in the tremor-dominant group (TD), and 58 in the mixed group (MT). No significant differences were recorded in plasma FAM19A5 levels among different exercise types. Further, log values for FAM19A5 levels were found to be smaller in the MT group as compared to AR and TD groups. However, these differences were found to be statistically insignificant, as assessed using *post-hoc* multiple comparison tests (*P* = 0.389 and *P* = 0.163, respectively) (Table 2).

**TABLE 2:** Comparison of three motor subtypes of PD.

	AR	TD	MT	Test value	*P*-value
Number	47	71	58	/	/
FAM19A5(log)	2.48 ± 0.58	2.52 ± 0.46	2.37 ± 0.50	*F* = 1.509	0.224
Gender (female/male)	(29,18)	(42,29)	(33,25)	χ^2^ = 0.248	0.883
Age (year)	65.81 ± 8.73	68.85 ± 9.01	66.78 ± 10.53	*F* = 1.615	0.202
Duration of illness (months)	24 (12,36)	24 (12,60)	33 (12,61.5)	*H* = 0.925	0.630
Age of onset (year)	63.08 ± 8.54	65.07 ± 10.08	62.97 ± 10.66	*F* = 0.907	0.406
Modified HY-stage	2(1.5,2.5)	2 (1, 2)	2 (1.38,2.5)	χ^2^ = 5.021	0.081
LEDD	337.5 (237.5,375)	337.5 (237.5,350)	337.5 (228.13,390.63)	χ^2^ = 0.568	0.753

### Expression of Plasma FAM19A5 in Non-motor Symptoms

On the basis of ROME III functional constipation criteria, 98 PD patients were included in the PD without constipation group, while 78 patients were included in the PD with constipation group. Although plasma FAM19A5 levels were found to be higher in PD with the constipation group (2.475 ± 0.479) as compared to PD without the constipation group (2.442 ± 0.547), these differences were statistically insignificant (*P* = 0.67, *t* = –0.472).

Further, based on the RBDSQ score, 62 PD patients were included in the PD with RBD group (2.447 ± 0.584) and 114 patients in PD without RBD group (2.462 ± 0.471). Most of the patients with RBD experienced insomnia, dreaminess, dream enactment behavior (DEB), and limb movement behaviors. Importantly, a Student’s t-test revealed that differences in the plasma FAM19A5 levels for the two groups were not statistically significant (*P* = 0.862, *t* = –0.174).

**FIGURE 5:**
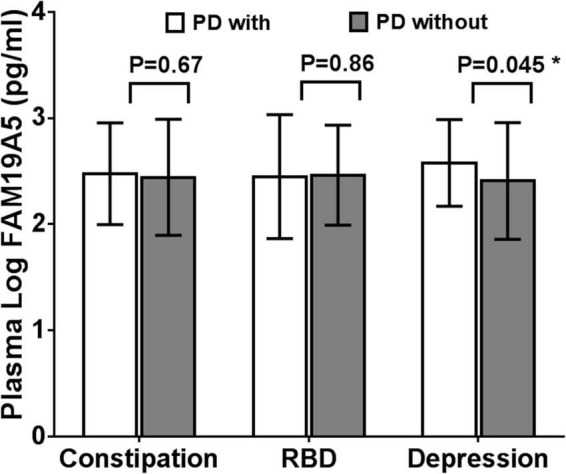
Expression of FAM19A5 in different non-motor symptoms. In constipation and RBD subtypes, log-transformed plasma levels of FAM19A5 were not significantly different between two subtypes. In depression subtype, FAM19A5(log) PD with depression was 2.576 ± 0.408 and PD without depression was 2.406 ± 0.549 (*P* = 0.045 < 0.05, *t* = −2.012). * indicates statistically significant values with *P* < 0.05.

Following this, the patients were further grouped on the basis of the PHQ-9 questionnaire score. In particular, 52 PD patients were included in the PDD subgroup and 124 patients in the PD without depression subgroup. A Student’s t-test showed that plasma FAM19A5 levels were significantly elevated in the PDD group (2.576 ± 0.408) as compared to PD without depression (2.406 ± 0.549) group (*P* = 0.045 < 0.05, *t* = −2.012) (Figure 5).

All these findings indicated that the plasma FAM19A5 levels were related to Parkinson’s depression. Following this, correlation analysis was carried out between plasma FAM19A5 levels and degree of depression in Parkinson’s patients, which showed a significant correlation at the level of 0.05. Interestingly, it was observed that PDD developed at an earlier age, which indicated that the disease in such cases would likely last longer. In addition to this, no differences were recorded in the depression factors, including gender, age, and phenotype of motor symptoms (Table 3).

**TABLE 3:** Comparison of PD with/without depression.

	PD with depression	PD without depression	Test value	*P*-value
Number	52	124	/	/
FAM19A5(log)	2.59 ± 0.39	2.41 ± 0.55	*t* = 2.146	0.033*
Gender (female/male)	33/19	71/53	χ^2^ = 0.916	0.339
Age (year)	65.17 ± 9.03	68.27 ± 9.60	*t* = –1.978	0.048*
Duration of illness (months)	36 (19.5,60)	24 (12,60)	*Z* = –1.745	0.081
Age of onset (year)	61.28 ± 10.22	64.92 ± 9.59	*t* = 2.253	0.026*
Modified HY-stage	2 ± 0.754	2.08 ± 0.761	*t* = 0.804	0.422
LEDD	337.5 (271.875,375)	337.5 (206.25,375)	*Z* = –1.100	0.271
**Phenotype of onset**				
Tremor-dominant	16 (30.8%)	31 (25%)	χ^2^ = 0.723	0.697
Akinetic-rigid	19 (36.5%)	52 (41.9%)		
Mixed	17 (32.7%)	41 (33.1%)		

* indicates statistically significant values with P < 0.05.

### Diagnostic Efficacy of Radiomics Combined With Plasma FAM19A5 for Prediction of Parkinson’s Disease

**FIGURE 6:**
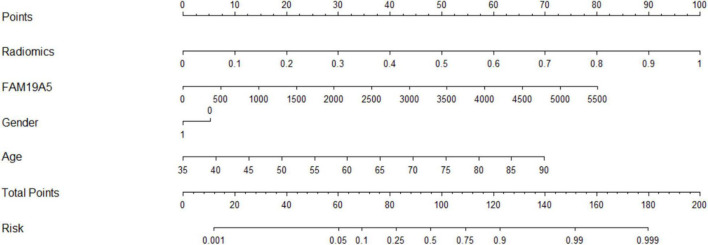
MRI-based radiomics and FAM19A5 nomogram for the prediction of PD.

The extracted and normalized radiomics signature of bilateral substantia nigra and red nucleus regions in MRI images were combined with log-transformed plasma levels of FAM19A5, gender, and age to develop a logistic regression model for the prediction of PD. Further, fivefold cross-validation was used to test the model performance. For each cross-validation, 164 subjects were randomly divided into two groups, namely training and validation groups, which included 131 and 33 subjects, respectively. The results showed that the logistic model exhibited excellent prognostic ability in predicting PD, with an accuracy of 85.6% (AUC = 0.913, 95% CI = 0.861–0.955) (Figure 3). The weightage of each risk factor was visualized using a nomogram (Figure 6).

### Diagnostic Efficacy of Imaging Omics Combined With Plasma FAM19A5 in Parkinson’s Disease Depression Subgroup

**FIGURE 7:**
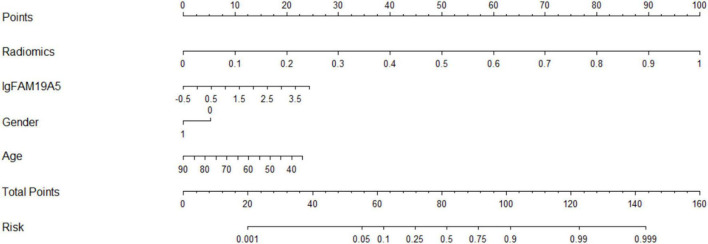
MRI-based radiomics and FAM19A5 nomogram for discrimination of PDD.

For depression subgroup analysis, logistic regression was performed using extracted and normalized radiomics signatures for the bilateral amygdala, head of the caudate nucleus, and putamen ROIs, obtained from MRI images, which were combined with log-transformed plasma levels of FAM19A5, gender, and age. The modeling procedure utilized fivefold cross-validation to test the performance of the resulting model. For each cross-validation, a total of 83 subjects were randomly divided into training and validation groups, which included 66 and 17 subjects, respectively. The results were recorded and visualized using ROC curves, and the area under the curve (AUC) was calculated. The results demonstrated the excellent performance of the logistic model in discriminating PD and PDD, with an accuracy of 87.8% (AUC = 0.937, 95% CI = 0.845–0.970) (Figure 4). The logistic weights for each risk factor were visualized using a nomogram (Figure 7).

## Discussion

In this study, the plasma levels of FAM19A5 were found to be significantly increased in PD patients. The present study is the first to assess FAM19A5 expression in PD patients. The study tried to explore the association between plasma FAM19A5 levels and some of the common non-motor symptoms observed in PD, wherein a correlation with PDD was reported. In particular, plasma FAM19A5 levels in the PDD group were recorded to be higher as compared to PD without depression group. Radiomics features and plasma FAM19A5 levels were further used to establish a PD prediction model, which effectively improved predictive performance to identify PD and PDD.

Family with sequence similarity 19 member A (FAM19A5) is a chemokine, which is expressed in the brain, optic nerve, and spinal cord. In particular, FAM19A5 exhibits a wider expression pattern throughout the CNS (Yushkevich et al., 2018; Lei et al., 2019). It can be secreted by glutamate neurons and neuroglia, which include microglia and astrocytes. These two types of cells constitute the innate immune cells of the brain. In recent years, many clinical and animal studies confirmed the role of neuroinflammation in PD. It has been previously shown that the CNS immune response interacts with the peripheral circulatory system immune response, which could further destroy the blood-brain barrier and induce peripheral lymphatic invasion. Neurotoxic factors released by microglia and astrocytes increase and aggravate inflammatory responses in the brain (Hirsch et al., 2012; Muldoon et al., 2013; Shao et al., 2015). Previous studies also reported evidence to support the current hypothesis that plasma FAM19A5 acts as a pro-inflammatory factor. In one of the studies, TNF-α was injected into the lateral ventricle of the mouse, wherein it mimicked the neuroinflammatory response and increased the expression of FAM19A5, IL-1b, IL-6, cyclooxygenase 2 (COX2), and mPGES-1 in the hypothalamus (Jha and Suk, 2013). In PD, TNF-α is known to bind to TNF-α receptor, present on the surface of microglia, to produce more pro-inflammatory factors, such as inducible nitric oxide synthase (iNOS), COX2, and IL-6 (Goetz et al., 2004; Lefkowitz and Lefkowitz, 2008; Hernandes and Britto, 2012; Kang et al., 2020), which may be one of the reasons for the increased plasma FAM19A5 levels in PD patients. However, none of the previously conducted meta-analysis studies reported any association of depression or its severity with IL-6, IL-1β, and TNF-α. It is previously known that demyelination occurs after brain injury, which leads to axonal degeneration and neuronal and glial cell death. In a previous study (Zhang et al., 2018), increased levels of FAM19A5 in oligodendrocyte precursor cells of traumatic brain injury-induced FAM19A5-LacZ KI mouse penumbra, which supported a positive role of neuroinflammation in the repair process during the early stages of PD (Shahapal et al., 2019). However, in the long-term pathological process, astrocytes and microglia were reported to get constantly activated, resulting in the production of glial scars and causing irreversible damage (Galli et al., 2019). Clarification of the underlying pathological mechanisms of FAM19A5 involved in the onset of PDD has important clinical significance. A larger multicenter study is warranted in the future to further confirm the correlation between the serum FAM19A5 levels and PD and PDD subjects.

Non-motor symptoms of PD are very common. In fact, these symptoms might appear before motor symptoms, suggesting their potential to be used as a means for early PD diagnosis. The most remarkable predictors of non-motor PD symptoms are constipation, anosmia, RBD, and depression. Among these, constipation usually occurs first (Schapira et al., 2017). The mechanism of action of the brain-gut axis in PD has been proposed in recent years. According to the proposed mechanism, the deposition of α-synuclein spreads from the intestine through the autonomic plexus to the brainstem, which further involves the cortex (Niranjan, 2013). The pathogenesis of RBD in PD patients might be related to dopaminergic defects in the substantia nigra, striatum, and brain stem nuclei, such as the locus coeruleus/subcoeruleus complex (Sommerauer et al., 2018; Ambrosini et al., 2019). This mechanism explains the pattern and sequence of non-motor symptoms. In this study, plasma FAM19A5 levels were found to be unrelated to constipation and RBD, which might be attributed to the absence of FAM19A5 expression in the intestinal tract (Li et al., 2017). However, in the present study, plasma FAM19A5 levels correlated with PD depression symptoms. In a previous study, Han et al. (2020) reported that MDD patients who did not receive drug treatment exhibited significantly higher FAM19A5 levels as compared to healthy controls. Moreover, the serum FAM19A5 levels in MDD patients were found to be negatively correlated with the thickness of the frontal prefrontal area, left posterior cingulate gyrus, right cuneate lobe, and prefrontal area. This study further indicated that FAM19A5 might be associated with neurodegeneration in brain regions involved in emotional processing. The pathogenesis of PDD is quite complex, and the pathophysiological mechanisms involved in this disease are not fully understood. One well-established factor is the deletion of dopamine in the substantia nigra-striatal system in PD patients. The decreased ability of glial cells to clear glutamate, induced by repeated chronic stress, might play a role in the pathogenesis of PD. This neurotoxicity further leads to a decrease in the density of glutamate neurons, γ-aminobutyric acid neurons, and acetyl cholinergic neurons in PDD patients, which ultimately leads to prolonged depression symptoms (Valentine and Sanacora, 2009; Wang et al., 2018).

The loss of dopamine neurons in the substantia nigra is a typical pathological feature of PD. It manifests as atrophy of the black volume in T2-weighted images. Han et al. (2020) previously showed that in patients with major depression, plasma FAM19A5 levels were significantly negatively correlated with gray matter volume of the prefrontal area, left posterior cingulate gyrus, and right cuneiform lobe. However, this study did not make predictions based on imaging features. In the current study, the prediction model established by combining radiomic features of MRI data, plasma markers, and white matter lesions demonstrated high accuracy in the prediction of PD. In a previous study, Xiao et al. (2019) proposed a method based on a convolutional neural network (CNN) with quantitative susceptibility mapping (QSM), which could distinguish PD and HC groups (accuracy: 0.85, AUC: 0.93), wherein 141 subjects were divided into PD and HC, with 88 and 53 patients, respectively. Dopamine is known to be involved in motor symptoms. Additionally, it also participates in the regulation of emotional activities. It has been previously reported that multiple DA transmission pathways get affected during PD, which leads to striatal-frontal and limbic system dysfunction. This in turn causes behavioral, emotional, and cognitive impairment. To study the changes in the nigrostriatal-limbic system in PD depression, 82 PD patients and 82 healthy controls were selected, and plasma FAM19A5 and structural MRI data were collected. Further, radiomics features were extracted from several representative ROIs (black, red nucleus, caudate nucleus head, and putamen). The AUC value for the logistic regression model, based on the combination of radiomic features and plasma marker, was found to be 0.913 (95% CI = 0.861–0.955), which indicated that the model could efficiently distinguish PD patients from controls. In addition to research on depression prediction in PD patients, a logistic regression model was trained using extracted and normalized radiomics signature of the bilateral amygdala, head of the caudate nucleus, and putamen regions in MRI images, and combined with plasma FAM19A5 level, gender, and age. The AUC value of the logistic regression model was recorded to be 0.937 (95% CI = 0.845–0.970), which indicated the good predictive ability of the model for the occurrence of depression in PD patients. The present study was associated with certain limitations. First, the study did not involve MR images for all participants, which might weaken the connection between plasma FAM19A5 and brain nuclei, and thus reduce the accuracy of the prediction model. In addition, recently have found that different fields have a strong impact on image texture eigenvalues and human exploration (Mayerhoefer et al., 2020). Although we did not differentiate between MRI of different field intensities, we randomly selected patients in the training and validation groups. The data in the validation group included 3-T and 1.5-T data, which proved that our model was effective for different field intensities.

In summary, the results of the present study suggested that increase in blood FAM19A5 levels might be related to neuroinflammation and neurodegeneration in PD patients. The study also reported that plasma FAM19A5 levels in PD patients were related to depressive symptoms. Thus, plasma FAM19A5 levels in combination with imaging omics could be effectively used for the prediction of PD and PDD.

## Data Availability Statement

The original contributions presented in the study are included in the article/Supplementary Material, further inquiries can be directed to the corresponding author/s.

## Ethics Statement

The studies involving human participants were reviewed and approved by Ethics Committee of the Affiliated Hospital of Qingdao University. The patients/participants provided their written informed consent to participate in this study.

## Author Contributions

X-nL, A-bM, and A-jM were major contributors in concept, design, the definition of intellectual content, and experimental studies. D-pH, M-jQ, and MZ performed the data curation. X-dP and A-jM supervised this research. All authors read and approved the final manuscript.

## Conflict of Interest

A-bM was employed by Shanghai Xunshi Technology Co., Ltd. as a data engineer and directly participated in data analysis of this study. This study did not receive any financial support from the company. The remaining authors declare that the research was conducted in the absence of any commercial or financial relationships that could be construed as a potential conflict of interest.

## Publisher’s Note

All claims expressed in this article are solely those of the authors and do not necessarily represent those of their affiliated organizations, or those of the publisher, the editors and the reviewers. Any product that may be evaluated in this article, or claim that may be made by its manufacturer, is not guaranteed or endorsed by the publisher.

## Footnotes

1
http://itksnap.org/

2
http://PyRadiomics.readthedocs.io/en/latest/
